# Recent Polygenic Adaptation in Heavily Fished Malawi Cichlids

**DOI:** 10.1111/mec.70443

**Published:** 2026-06-22

**Authors:** Alexander Hooft van Huysduynen, Francisco Campuzano Jiménez, Julia Camacho Garcia, Gudrun De Boeck, Bosco Rusuwa, Hannes Svardal

**Affiliations:** ^1^ Evolutionary Ecology Group, Department of Biology University of Antwerp Antwerp Belgium; ^2^ ECOSPHERE, Department of Biology University of Antwerp Antwerp Belgium; ^3^ School of Natural and Applied Sciences University of Malawi Zomba Malawi; ^4^ Naturalis Biodiversity Center Leiden the Netherlands

## Abstract

Intense fishing pressure can drive rapid evolution in wild populations, yet the underlying genomic mechanisms often remain elusive. Here, we investigate the genomic consequences of five decades of intense harvesting on the cichlid fish *Copadichromis mloto* in Lake Malombe, Malawi. By comparing whole‐genome data from almost 200 individuals from the heavily fished Lake Malombe with less‐fished populations in Lake Malawi, we identified a widespread signal of recent, population‐specific positive selection. This genomic footprint, spanning hundreds of loci, is consistent with a rapid, polygenic adaptive response. The candidate genes under selection were significantly enriched for high‐level developmental processes, including neurogenesis and the formation of the heart and muscle, providing a plausible mechanistic link to the dramatic reduction in size and age at maturity observed in this population. Furthermore, our strongest candidate loci include genes with known roles in controlling somatic growth (*zdhhc13*, *myh7, csrp3, gnb1l*) and reproductive timing (*mpped2a*, *kitlga, kat6a, stra6*). We also found that candidate genes were nearly five‐fold more likely to be differentially expressed in male gonad tissue, providing a strong link between selection and regulatory changes in a key reproductive organ. Our findings point towards a polygenic basis for fisheries‐induced evolution in this tropical system and highlight how intense harvesting can rapidly reshape the genetic architecture of complex life‐history traits, with important implications for the long‐term resilience of exploited fish stocks.

## 1 Introduction

Human‐induced environmental changes can impose strong selective pressures that drive rapid evolution in wild populations, ultimately determining whether they adapt or face extinction (Allendorf and Hard 2009; Hendry et al. 2017). While such contemporary evolutionary responses are now widely documented across many species (Baltazar‐Soares et al. 2021), the underlying molecular mechanisms remain largely unknown (Hutchings and Kuparinen 2021; Pelletier and Coltman 2018). A critical challenge is to move beyond observing phenotypic shifts and identify the specific genetic loci that facilitate adaptation to anthropogenic stress. Uncovering this genetic basis is fundamental for distinguishing heritable evolutionary change from phenotypic plasticity and for building a mechanistic understanding of how populations respond to anthropogenic pressures.

Classic examples of rapid evolutionary responses to human activities include the spread of industrial melanism in peppered moths (Hof et al. 2016), the reduction of tusk size in elephants due to ivory poaching (Campbell‐Staton et al. 2021), and decreased horn size in bighorn sheep targeted by trophy hunting (Pigeon et al. 2016). Perhaps the most widespread example is fisheries‐induced evolution (FIE), which describes shifts in the life‐history traits of commercially harvested fish. This phenomenon has been documented across diverse aquatic systems and taxa, with compelling field, experimental and theoretical evidence demonstrating that intense fishing pressure selects for traits like earlier maturation at a smaller size (Conover and Munch 2002; Heino et al. 2015; Kuparinen and Festa‐Bianchet 2017).

Despite strong evidence for phenotypic change, identifying the genetic basis of FIE has proven exceptionally challenging (Han et al. 2025; Hutchings and Kuparinen 2021). This knowledge gap limits our understanding of the molecular pathways driving these rapid life‐history shifts and understanding of whether they represent heritable adaptation or phenotypic plasticity. For instance, a temporal genomic study of Atlantic cod, a classic example of FIE, found no evidence of strong, fisheries‐driven genomic change (Pinsky et al. 2021). The authors suggested that the observed life‐history shifts were likely due to highly polygenic adaptation or plastic responses, rather than selection on a few major‐effect loci. This highlights the complexity of FIE, where confounding factors such as changes in population density, climate or predation may also contribute significantly to phenotypic shifts (Hutchings and Kuparinen 2021).

While most studies of fisheries‐induced evolution have focused on marine systems or temperate fish species, tropical freshwater taxa offer a vital but understudied context for this research (Heino et al. 2015). The cichlid fishes of Lake Malawi provide a compelling system to investigate rapid, human‐driven evolutionary change. Fisheries in Lake Malawi and its peripheral lakes are a cornerstone of the regional economy, constituting 40% of all dietary animal protein and 4% of Malawi's national GDP (M'balaka et al. 2024). However, this vital resource is under threat, as many of the lake's over 800 cichlid species (Konings 2016) have experienced dramatic population declines due to intense artisanal and commercial fishing (M'balaka et al. 2024; Turner 1995). Species in shallow habitats have faced increasing fishing pressure over the last five decades, creating a strong selective landscape. This pressure is not uniform, varying substantially across the range of many species and reaching its most extreme intensity in Lake Malombe.

**FIGURE 1:**
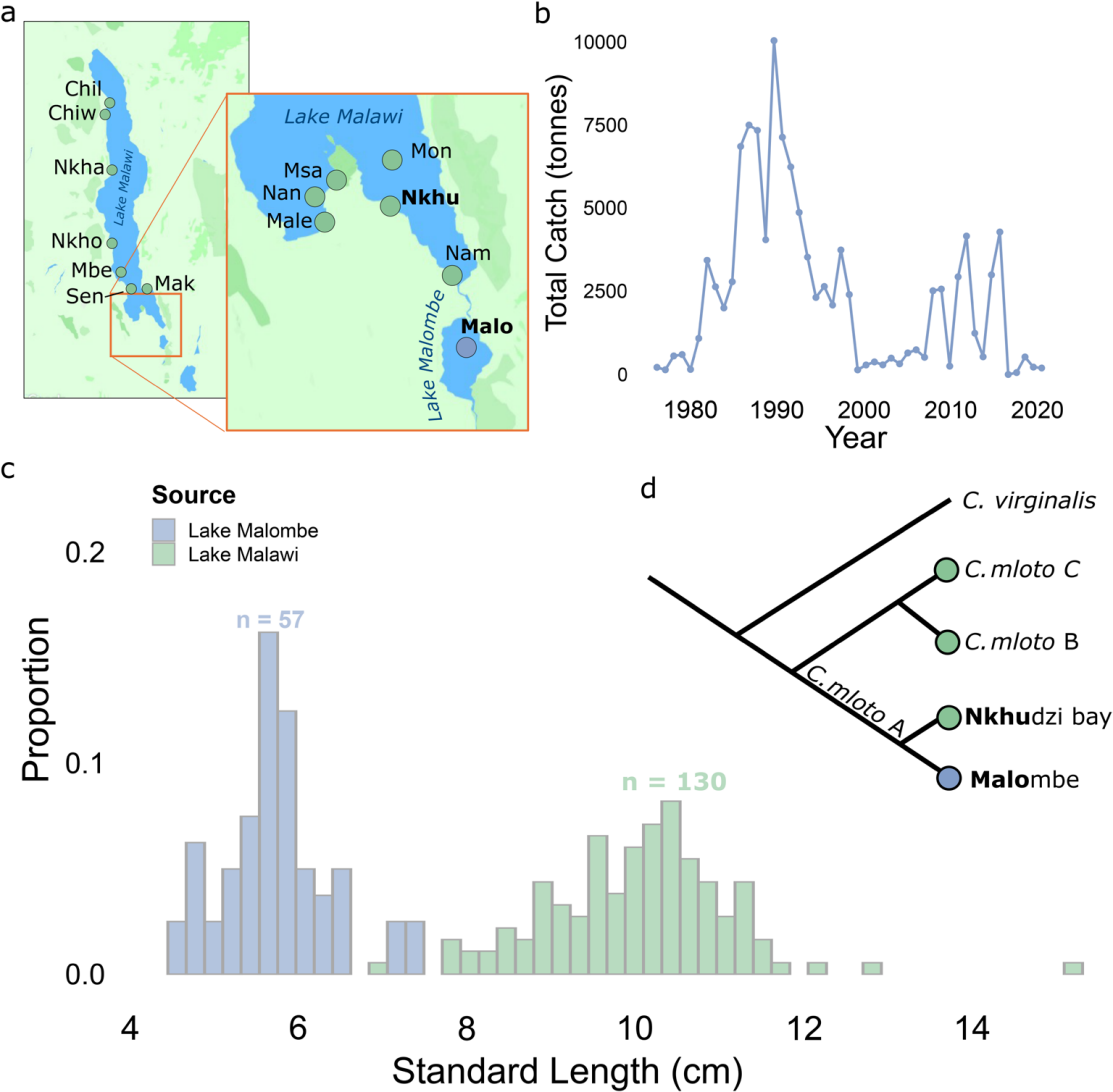
Geographic, ecological and phylogenetic context of the study populations. (a) Map showing geographic context and sampling locations in Lake Malawi (green points) and Lake Malombe (blue point) used in this study. Denoted sampling locations are specified as follows: Chilumba (Chil), Chiweta (Chiw), Nkhata Bay (Nkha), Nkhotakota (Nkho), Mbenji_Islands (Mbe), Senga Bay (Sen), Msaka (Msa), Nankhwali trawler (Nan), Malembo (Male), Monkey Bay (Chenga trawler) (Mon), Nkhudzi Bay (Nkhu), Namiasi‐Palm Beach (Nam), Lake Malombe (Malo) (b) Temporal trend in total annual catch from Lake Malombe fisheries of haplochromine cichlids, data from: Makwinja et al. (2021). (c) Standard length distributions of *Copadichromis mloto* from field samples collected in Lake Malombe (blue bars) and Lake Malawi (green bars) across several field expeditions since 2016. (d) Scheme of phylogenetic relationships of *Copadichromis mloto* complex, including clades A, B, C as defined in Sawasawa et al. (2024).

Lake Malombe is a young (< 200 years old), shallow and highly productive water body at the outflow of Lake Malawi (Figure 1a), making it particularly vulnerable to environmental change. In the 1980s, the introduction of purse seine fishing (locally called ‘Nkatcha’) led to extremely intense and indiscriminate harvesting of fish of all sizes. This practice first caused the collapse of the local tilapia (‘Chambo’) fishery around 1990 and has since been directed at the lake's diverse haplochromine cichlids, threatening many with extinction (Hara et al. 1999; Weyl et al. 2004; Zwieten et al. 2011). While Nkatcha fishing was restricted under fisheries regulations in Lake Malawi and is largely impractical across most of the lake's greater depths, it remained prevalent in the shallow waters of Lake Malombe (Makwinja et al. 2021; Weyl et al. 2004; Zwieten et al. 2003). The direct consequence of this method has been a stark decline in fish stocks; despite increasing fishing effort, total catch biomass plummeted from a peak of ~10,000 t per year in 1990 to less than 1000 t per year in the early 2000s and a stunted recovery since (Figure 1b) (Makwinja et al. 2021; Zwieten et al. 2003).

To investigate the genomic consequences of this intense harvest, we focused on *Copadichromis mloto* , a species of high market value that used to be abundant and economically significant in the fisheries of both Lake Malawi and Lake Malombe. Coinciding with the increase in fishing pressure in Lake Malombe, the local *Copadichromis mloto* population has shown a strong decline in relative abundance and a marked reduction in size and age at maturity (see Figure 1c) (Makwinja et al. 2021; Tweddle et al. 1995). This phenotypic shift, consistent with males reproducing in their first year compared to in their third year in Lake Malawi (Iles 1971), represents a classic signature of FIE, whereby high mortality selects for earlier maturation at a smaller body size. These observations strongly suggest that the Lake Malombe population of *C. mloto* has been under intense selection. Therefore, in this study, we aim to identify genomic regions under recent selection and highlight candidate genes plausibly involved in these critical life‐history shifts. By comparing whole‐genome and transcriptome data from Lake Malombe with that of less intensely fished populations from Lake Malawi, we investigate the genomic basis of this apparent rapid adaptation.

## 2 Materials and Methods

### 2.1 Sample Collection and Sequencing

*Copadichromis mloto* (*N* = 224) were obtained from local fishers at landing sites across southern Malawi during expeditions in 2002, 2016 and 2017. For genomic analysis, a right pelvic fin was excised from each individual, preserved in absolute ethanol, and stored at −80°C. Whole‐body voucher specimens were also collected, fixed in 10% formalin, preserved in ethanol, and are housed at the Royal Museum for Central Africa, Belgium (2002 collection) and the Museum of Zoology, University of Cambridge, UK (2016–2017 collections). Samples have previously been included in Sawasawa et al. (2024) and assigned to one of three *C. mloto* clades (A, B, C) or re‐classified as *C. virginalis* . DNA was extracted from fin clips using standard protocols and short reads (100 to 150 bp paired‐end) were obtained on Illumina HiSeq platforms as described in Sawasawa et al. (2024) and Blumer et al. (2025) reaching genomic coverages between 7.2× and 36.8× (median of 14.5×) (Table S1).

### 2.2 Alignment and Variant Calling

Sequencing reads were mapped to the *Astatotilapia calliptera* reference genome (fAstCal1.2, accession GCA_900246225.3, https://www.ncbi.nlm.nih.gov/assembly/GCF_900246225.1) using BWA‐MEM (version 0.7.17; Li 2013). *C. mloto* & *A. calliptera* belongs to the Malawi haplochromine cichlid radiation and these species share low sequence divergence of ~0.096% (Malinsky et al. 2018) comparable to within human diversity (0.075%–0.1%; 1000 Genomes Project Consortium 2015). Mean mapping rates of 98.38% across samples (range: 97.4%–98.9%) are consistent with values reported for within‐radiation cichlid studies, indicating that cross‐species mapping artefacts are unlikely to substantially confound our results (Malinsky et al. 2018). Variant calling was conducted with bcftools (v1.14; Danecek et al. 2021). To ensure high‐quality variant calls, sites were masked under the following conditions: if more than 10% of mapped reads had zero mapping quality, if the overall mapping quality was below 50, if mapping quality was significantly biassed between strands (*p* < 0.001), or if total read depth across all samples was either above the 97.5th percentile or below the 2.5th percentile. Additionally, heterozygous sites were filtered out when a binomial test indicated a significant allele balance bias (PHRED > 20). Positions with evidence of excess heterozygosity (Inbreeding Coefficient < 0.2) or with more than 20% missing data were also excluded. Only single nucleotide polymorphisms (SNPs) were retained for further population genetic analyses. Following these filtering steps, the dataset included approximately 41.7 million biallelic SNPs spanning 22 chromosomes. The variant call format (VCF) file of whole‐genome biallelic SNP dataset is available on Zenodo: (https://doi.org/10.5281/zenodo.17657791). For the selection statistics analysis below, the *C. mloto* callset was complemented by population SNP variation data from 45 additional species from Blumer et al. (2025) (Table S2).

### 2.3 Genome Wide Selection Statistics

Tajima's *D* was calculated in 10 kb windows for all individuals of each defined population with vcftools (v0.1.16; Danecek et al. 2011). A normalized Fay and Wu's *H* calculation (Zeng et al. 2006) derived from the allele frequency spectra of each population was calculated in a custom python script available on Zenodo (https://doi.org/10.5281/zenodo.17649119).

### 2.4 Genome Scans for Malombe‐Specific Signals of Selection

To identify Malombe‐specific allele frequency shifts we used the Population Branch Statistic (PBS) developed by Yi et al. (2010) based on the per SNP pairwise *F*_ST_ values of the Malombe, Nkhudzi bay and Namiasi Palm beach populations (‘Malo’, ‘Nkhu’ & ‘Nam’, respectively in Figure 1a). PBS gives an estimate of *F*_ST_ differentiation since the last common ancestor of Malombe and sister population Nkhudzi Bay (Figure 1d) allowing us to determine SNP differentiation unique to the Malombe population. The Namiasi Palm Beach population is used as an outgroup in the PBS calculation, which despite its geographic proximity to Lake Malombe phylogenetically belongs to the *C. mloto* B sister clade (Figure 1d; Sawasawa et al. (2024)). Weir and Cockerham (1984) *F*_ST_ values were estimated using vcftools (v0.1.16; Danecek et al. 2011).

To detect hard and soft selective sweeps across the genome we explored the haplotype homozygosity in the Malombe population around each SNP using the software *H*‐scan (version 1.3, downloaded from: https://messerlab.org/resources/; Schlamp et al. 2016). We performed *H*‐scan on the Malombe population and the Nkhudzi bay populations and increased the specificity for Malombe‐specific homozygosity patterns by subtracting the observed *H*‐statistic of the Nkhudzi bay population from the *H*‐statistic estimated for the Malombe population. This modification isolates the selective sweep signals that are unique to the Malombe population (i.e., occurred after its divergence from the Nkhudzi bay population).

To integrate complementary signals of recent selection, we computed a Fisher's Combined Score (FCS) per SNP by combining the percentile ranks of the PBS and the normalized *H*‐value from *H*‐scan, following Choin et al. (2021). For each SNP, we computed the negative base‐10 logarithm of the percentile rank for PBS and *H* separately, then summed these to obtain per‐SNP FCSs. This method prioritizes SNPs with strong rank‐based signals in both selection scans while reducing dependence on raw magnitudes.

### 2.5 Neutral Demographic Simulations

To evaluate the statistical significance of observed selection scan results, we estimated a distribution of the FCS statistic under a neutral demographic scenario using msprime (v1.3.3; Baumdicker et al. 2022). Specifically, we simulated three‐population genealogies under the standard coalescent and superimposed neutral mutations to produce VCF files that were later processed identically to empirical data. Model parameters were based on genome‐wide estimates derived from our dataset and used recombination and mutation rate estimates from earlier studies on Malawi cichlids (Malinsky et al. 2018; Talbi et al. 2024). Effective population sizes were estimated from *π* (nucleotide diversity) using vcftools ‐‐window‐pi and the relation Ne = *π*/(4 *μ*), with a mutation rate of *μ* = 3.5 × 10^−9^. Recombination rate was fixed at a genome‐wide average of 6.17 × 10^−9^, based on linkage map data for *A. calliptera* (Talbi et al. 2024). The mutation rate used in simulations was 3.5 × 10^−9^, as previously estimated in Malawi cichlids (Malinsky et al. 2018). Split times were derived by subtracting the average within species coalescent times from between species coalescent times. Simulations were run over 1 Mb windows and summary statistics (PBS, H, FCS) were calculated per SNP for comparison with empirical values. Although residuals were consistently small (< 0.06 FCS points) across most of the matched percentile between the simulated and empirical FCS distributions, the upper tail showed a pronounced deviation towards higher empirical values (See Results: Figure 3c and Figure S1). This deviation started at an FCS of 5.512, corresponding approximately to the 99.99th percentile of the empirical values, and increased to an extreme residual exceeding 2.5 FCS points. Based on this trend we established the FCS value of 5.512 as an empirical selection threshold in further analyses. Scripts and configuration files for demographic simulations are available on Zenodo (https://doi.org/10.5281/zenodo.17649119).

### 2.6 Functional Annotation and Enrichment

To assess the potential functional consequences of selection on specific variants, we annotated all variants surpassing the empirical selection threshold with functional effect categories using SnpEff (v5.2.1; Cingolani et al. 2012) based on an annotated genome of *A. calliptera* (fAstCal1.2, ensemble release 105; Dyer et al. 2025) which contains 28,001 coding‐genes. Where ensembl stable IDs had not yet been assigned a gene name in this release, the name resolved in the updated fAstCal1.3 annotation (Ensembl release 112) is given in parentheses.

To determine whether the observed proportions of functional annotations (e.g., synonymous, non‐synonymous, etc.) differed from expectation, we computed an empirical distribution of annotation effects in minor allele frequency (MAF)‐matched set of SNPs using a custom Python script (script available on Zenodo: https://doi.org/10.5281/zenodo.17649119). For each SNP surpassing the selection threshold, control SNPs of similar MAF were randomly sampled from the full SNP dataset, over 1000 iterations. Significant overrepresentation in the proportions of functional annotation categories was assessed using binomial tests; *p*‐values were corrected for multiple testing using Benjamini‐Hochberg false discovery rate (FDR) considering results significant at FDR < 0.05.

Gene Ontology enrichment analysis was performed on genes located within candidate regions, defined as genomic regions extending ±25 kb around each locus under selection, reflecting the typical range of linkage disequilibrium in Malawi cichlids. Genes located within these regions were considered candidate genes. We conducted a Gene Ontology (GO) enrichment analysis using the topGO package (v2.38.1; Alexa and Rahnenfuhrer 2023) in R/Bioconductor (www.bioconductor.org). As a background, we used all annotated protein‐coding genes from the *A. calliptera* reference genome (fAstCal1.2, ensemble release 105, Dyer et al. 2025) and mapped to GO term assignments of *Dania rerio* (org.Dr.eg.db, v3.19.1). In total, 11,522 genes were successfully mapped to GO terms within the Biological Process (BP) ontology. The minimum category size was set to five genes (nodeSize = 5). Enrichment significance was evaluated using Fisher's exact test implemented in the *weight* algorithm of topGO (‘weightCount’ class). This algorithm improves interpretability by accounting for hierarchical relationships among GO terms (Alexa et al. 2006). Reported *p*‐values (Table S3) are not explicitly adjusted for multiple testing, but the authors note that for the topology aware algorithm used, *p*‐values can be regarded as effectively corrected, as the methods account for GO‐term dependencies (Alexa and Rahnenfuhrer 2023).

### 2.7 Differential Gene Expression Analysis

Tissue samples were collected from five tissues (brain, gill, gonad, liver, muscle) from six *C. mloto* males in breeding coloration from Msaka (Lake Malawi) and six males in breeding coloration from Lake Malombe. Differential expression analyses were restricted to males in breeding coloration to minimize expression variation associated with sex or reproductive state that would confound differential expression analyses across populations. Samples were stored in RNAlater at −80°C, then homogenized in Trizol with zirconia/silica beads. RNA was extracted using the Direct‐Zol RNA Purification Kit (Zymo Research), and quality assessed with Qubit and Tapestation (Agilent). RNA libraries (75 bp paired‐end) were sequenced at the Wellcome Sanger Institute on an Illumina HiSeq 4000. RNA‐seq was performed on a subset of 12 individuals also included in the whole genome sequencing dataset (Table S1); RNA and DNA library preparation were conducted independently, ensuring no shared technical biases between analyses.

The differential gene expression analysis comparing patterns of expression between the two populations was conducted using the RASflow pipeline (Zhang and Jonassen 2020). RNA‐seq reads were aligned to the *Astatotilapia calliptera* reference genome (fAstCal1.2) using HISAT2, and gene‐level counts were obtained using featureCounts (Liao et al. 2014). Differential expression analysis was conducted with PyDESeq2 (Muzellec et al. 2023), the Python implementation of DESeq2, applying log2 fold change > 1 and FDR‐adjusted *p*‐value < 0.01 as significance thresholds. To test whether differentially expressed genes were significantly enriched among genomic candidate genes from selection scans, we quantified pairwise overlaps and compared them to expectations under a random model using a hypergeometric test. For each DE gene list and selection analysis candidate gene set we compared the observed number of overlapping genes (*O*) to the expected overlap under random association (*E = A × B/N*) where *A* and *B* are the sizes of the two gene sets being compared and *N* is the total number of genes tested. Enrichment is expressed as an observed/expected (*O/E*) ratio and *p*‐values were corrected for multiple testing using the Benjamini‐Hochberg FDR procedure.

## 3 Results

### 3.1 Recent Positive Selection in the Lake Malombe Population

**FIGURE 2:**
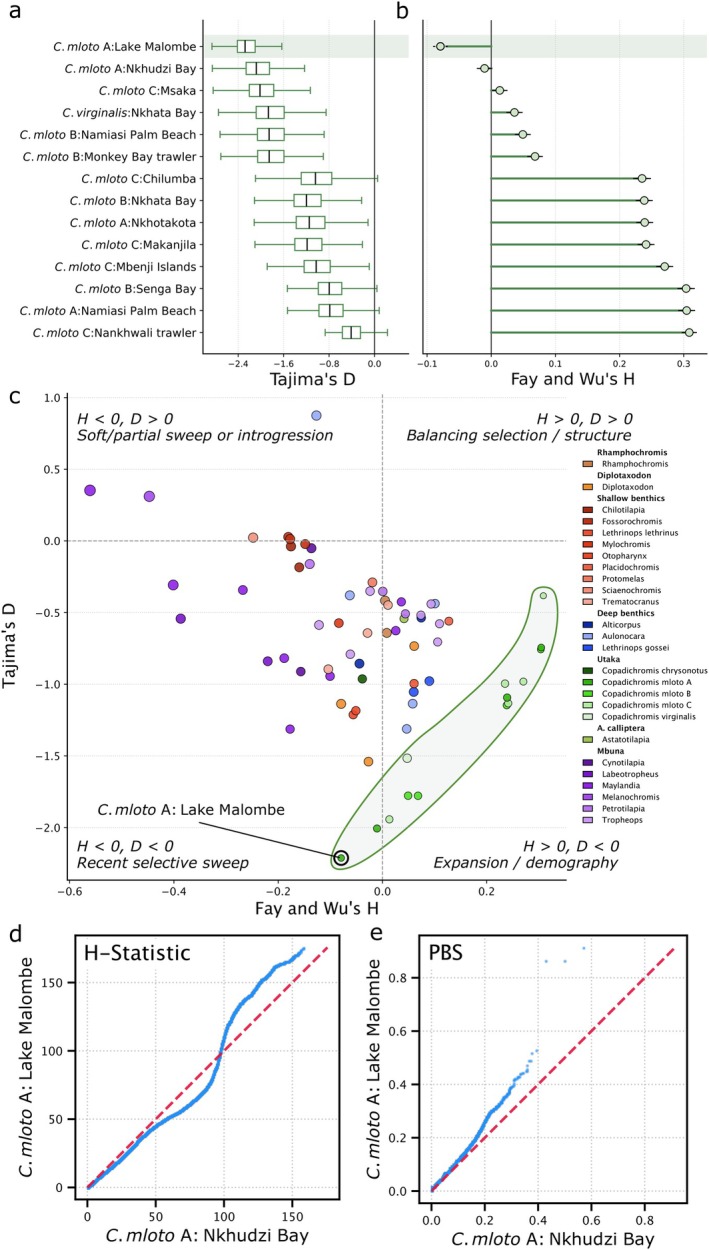
Genome‐wide summary statistics and selection signals in *Copadichromis mloto* (a, b) Distributions of Tajima's *D* (a) and Fay and Wu's *H* (b) across populations of the *C. mloto* / *C. virginalis* complex. Clades A, B and C are denoted as defined in Sawasawa et al. (2024). (c) Genome‐wide Tajima's *D* and Fay and Wu's *H* values in a broader phylogenetic context of Malawi cichlids. Genera are grouped by clade as defined in Blumer et al. (2025). Populations of the C. mloto complex are highlighted in green, with the Lake Malombe population annotated. (d, e) Quantile–quantile (Q–Q) plots comparing the Lake Malombe and Nkhudzi Bay *C. mloto* populations for the distributions of the *H*‐statistic (d) and Population Branch Statistic (PBS; e).

To investigate the evolutionary forces shaping the heavily fished Lake Malombe population, we first analysed genome‐wide allele frequency spectra for all *C. mloto* populations. We found that all populations exhibited negative mean values for Tajima's D, indicating a general excess of rare variants. While consistent with either population expansion or positive selection, the Lake Malombe population showed the most negative Tajima's *D* value, suggesting it has experienced the strongest demographic or selective event (Figure 2a).

To disentangle these two possibilities, we calculated Fay and Wu's *H* (*H*_FW_), a statistic that detects the excess of high‐frequency derived alleles characteristic of recent selective sweeps. In this analysis, the Lake Malombe population was a clear outlier; it was the only *C. mloto* population to show a strongly negative *H*_FW_ value (Figure 2b). To place this result in a broader phylogenetic context, we reanalyzed SNP data from Blumer et al. (2025), computing Tajima's *D* and *H*_FW_ for all cichlid populations with at least five sequenced individuals (Figure 2c). While Tajima's *D* and *H*_FW_ values varied considerably across species, the Lake Malombe population remained a notable outlier: it showed the most negative Tajima's *D* value among all species and populations and a significantly negative *H*_FW_, despite belonging to the species with overall most positive *H*_FW_ values.

In summary, the combination of a negative Tajima's *D* with a negative *H*_FW_ specific to the Malombe population of *C. mloto* provides compelling evidence that recent positive selection, in the form of pervasive selective sweeps, has occurred in the population since its divergence from the Lake Malawi populations.

### 3.2 Elevated Signatures of Selection Across the Malombe Genome

To further confirm that the Lake Malombe *C. mloto* population has been subject to recent, lineage‐specific selection, we performed genome‐wide selection scans using the Population Branch Statistic (PBS) and *H*‐scan. Quantile‐quantile plots showed that the Lake Malombe population had globally elevated values for both selection statistics compared to identical analyses for its sister‐population from Lake Malawi's Nkhudzi Bay (Figure 2d,e). This indicates that, on average, allele frequency differentiation and haplotype lengths are greater in the Malombe population. This genome‐wide pattern provides additional support for the conclusion that positive selection has been more pervasive or intense in the Lake Malombe lineage following its split from Lake Malawi populations. More critically, the extreme upper tail of both distributions was highly divergent between the sister populations, with higher values in the Malombe population. This extreme tail of the distribution corresponds to the specific genomic regions expected to have experienced the strongest selection.

### 3.3 Genomic Regions Under Malombe‐Specific Selection

To pinpoint specific genomic regions under recent selection in the Lake Malombe population, we combined population differentiation (PBS) and Malombe‐specific haplotype homozygosity (*H*‐scan) statistics into a Fisher's Combined Score (FCS), allowing us to detect loci with both Malombe‐specific allele frequency shifts and signatures of selective sweeps (see Materials and Methods for details).

**FIGURE 3:**
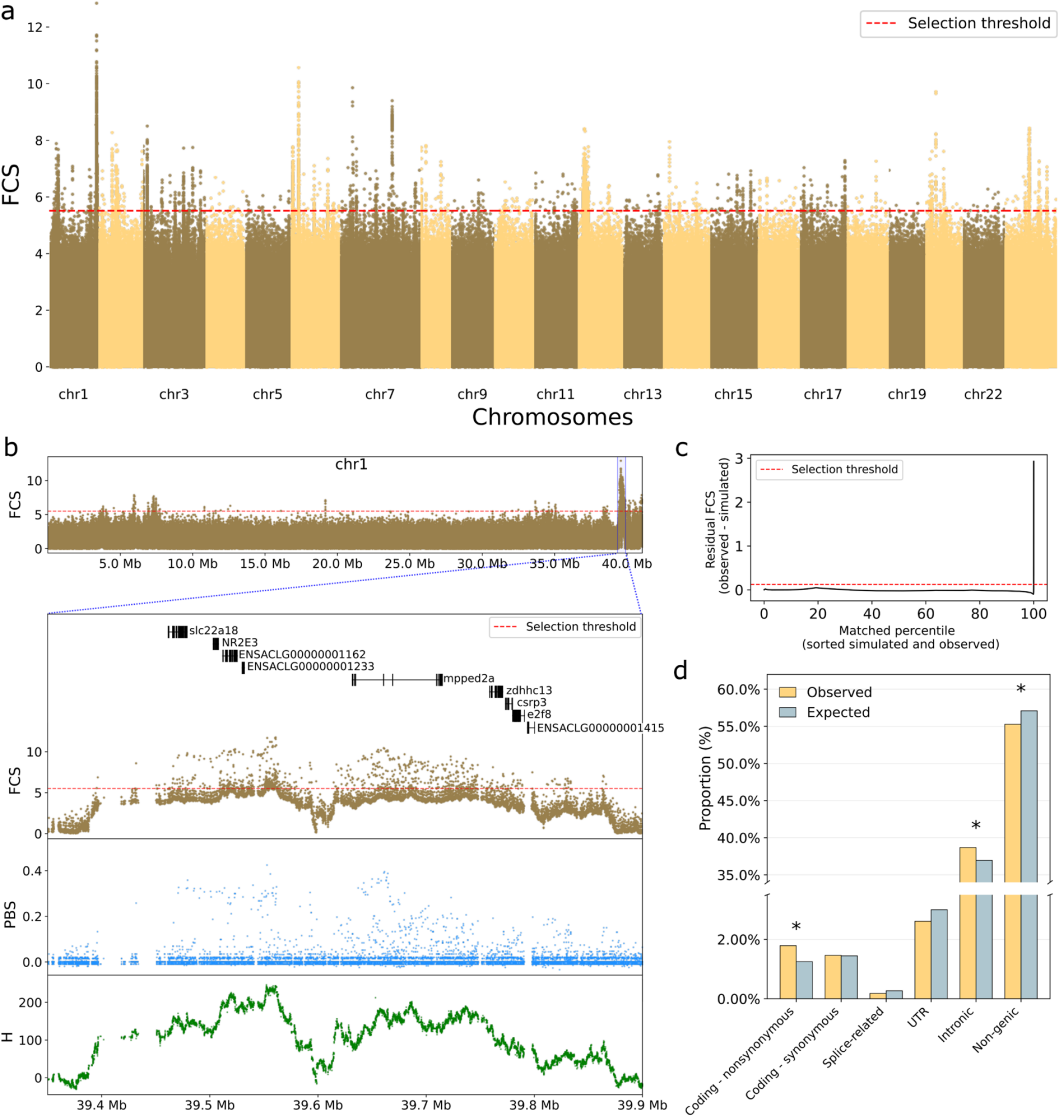
Characterization of selection signals in the Lake Malombe population. (a) Manhattan plot showing positive values of Fisher's Combined Score (FCS) per SNP across the genome. The dashed red line indicates the empirical selection threshold derived from neutral demographic simulations (FCS = 5.51). (b) Detailed view of the strongest selection peak on chromosome 1, showing positions of gene exons and symbols, as well as the SNP score for FCS, Population Branch Statistic (PBS) and *H* statistic (*H*). (c) Residual FCS value (observed—simulated) plotted against the ranked/matched simulated and empirical FCS value. The dashed red line along *y* axis indicates the threshold surpassed in the 99.99% percentile of empirical observations. (d) Bar plot showing proportional distributions of functional categories among SNPs surpassing the empirical selection threshold, and expectation from minor allele frequency (MAF) matched resampling. Asterisks (*) denote categories with significant overrepresentation (FDR < 0.05, binomial test).

A genome‐wide scan of FCS values revealed numerous outlier regions across the genome (Figure 3a). Among these signals, ten major peaks stood out as the most prominent candidates for recent, strong selection. Each peak corresponds to a genomic region extending approximately 25 kb on either side of clustered outlier SNPs. These ten regions collectively encompass 24 annotated genes, namely; *slc22a18, nr2e3, ENSACLG00000001162 (stra6), ENSACLG00000001233 (islr2), mpped2a, zdhhc13, csrp3, ENSACLG00000016851, ank2, ENSACLG00000009480, ENSACLG00000013470, si:dkey‐12e7.4, ENSACLG00000021888 (cnksr1), ENSACLG00000021917 (myh7), ENSACLG00000022020, ENSACLG00000022038 (nlrc5), kitlga, kat6a, ENSACLG00000003998, tbx1, gnb1l, ENSACLG00000024047, magi1, ENSACLG00000008501 (si:ch211‐108d22.2)*. Detailed plots showing the Fisher's Combined Score (FCS), *H*‐score and PBS for the highest peak on Chromosome 1 are presented in Figure 3b (see Figures S2–S10 for other major peaks). Intriguingly, functional annotations for genes among these ten major peaks reveal their involvement in key developmental processes, including somatic growth, muscle development and the timing of reproductive maturation (see Discussion).

### 3.4 A Widespread Signal Consistent With Polygenic Adaptation

While the most prominent peaks highlight major targets of selection, a key question is what proportion of the genome shows evidence of adaptation. To address this and identify a comprehensive set of candidate SNPs, we established a significance threshold that separates signals of positive selection from neutral background variation. We achieved this by comparing our empirical FCS distribution to a null distribution generated from neutral demographic simulations (see Material and Methods), which allowed us to define a stringent ‘empirical selection threshold’ at FCS > 5.51 corresponding to the top 99.99% of observations (Figure 3c; Figure S1).

A total of 4185 SNPs, located within 473 genomic regions, surpassed this threshold (Figure 3a). The widespread genomic footprint of outlier regions is consistent with a polygenic adaptation model, where selection acts on many loci across the genome. Further supporting the functional relevance of the outlier SNPs, we found that they were significantly enriched for non‐synonymous (amino acid changing) mutations but not enriched for synonymous mutations, indicating that selection has targeted functional variants (Figure 3d). Intronic variants were also significantly enriched among candidate selection SNPs, consistent with selection acting on cis‐regulatory variation and suggesting that gene expression changes contribute to the adaptive response alongside protein‐level divergence (Figure 3d).

### 3.5 Candidate Genes Are Enriched for Differential Expression Across Tissues

**FIGURE 4:**
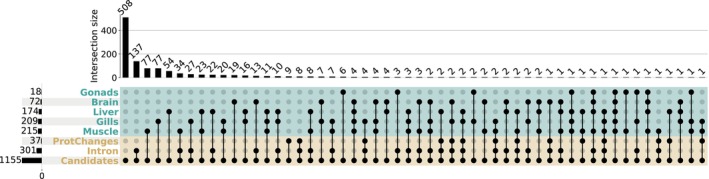
Overlap plot illustrating intersections among 1155 candidate genes. Candidate genes have transcription start sites within ±25 kb of SNPs surpassing the empirical selection threshold. Differentially expressed genes between Lake Malombe and Lake Malawi populations in five tissues. The ‘Intron’ category represents the subset of candidate genes containing at least one SNP in an intron region surpassing the empirical selection threshold. The ‘ProtChanges’ category represents the subset of candidate genes containing at least one non‐synonymous SNP surpassing the empirical selection threshold. Bars indicate the size of each intersection, and connected dots denote shared gene sets across tissues or categories.

To test whether candidate regions were also associated with regulatory changes, we performed a differential expression analysis between Lake Malombe and Lake Malawi individuals across five tissues (see Table S4 for differential expression results for each tissue). Although differential expression and non‐synonymous sequence changes represent distinct molecular outcomes, overlap between them can reveal genes where regulatory and protein‐level changes converge and provides stronger evidence for compounded involvement in adaptive divergence. Candidate genes were significantly more likely to be differentially expressed than non‐candidate genes in all tissues tested (Table 1, Figure 4). Notably, this enrichment was strongest in gonad, muscle and liver tissue, where candidate genes were between 4.4 and 4.7‐fold more likely to show differential expression than expected by chance (Table 1, Table S5). This strong link between signatures of selection and gene expression changes, particularly in the gonads, provides functional support for the role of these loci in the adaptive divergence of life‐history traits in the Lake Malombe population.

**TABLE 1:** Intersection between genes identified as candidates for selection in genome scans (‘All Candidates’) and the subset of genes containing SNPs surpassing the empirical selection threshold in intronic regions (‘Intronic’) and non‐synonymous sites (‘ProtChanges’) with differentially expressed (DE) genes detected in each tissue. Columns indicate the number of genes per set (Selection candidates and DE genes), the number of overlapping genes (Overlap) and the number expected by chance based on random resampling (Expected).

Selection candidate gene subset	DE tissue	# Selection candidate genes	# DE genes	Overlap	Expected (CI: low‐High)	Fold	Adjusted *p*
All candidates	Gonads	1155	197	18	3.8 (1–8)	4.74***	3.3e‐05
All candidates	Muscle	1155	4544	215	48.5 (37–61)	4.43***	3.3e‐05
All candidates	Liver	1155	3620	174	39.6 (29–51)	4.39***	3.3e‐05
All candidates	Brain	1155	1428	72	20.7 (13–29)	3.48***	3.3e‐05
All candidates	Gills	1155	3821	209	59.7 (47–73)	3.50***	3.3e‐05
Intronic	Gonads	301	197	5	1.0 (0–3)	5.14**	0.0028
Intronic	Brain	301	1428	27	2.2 (1–10)	5.10***	3.3e‐05
Intronic	Liver	301	3620	54	10.8 (5–17)	4.99***	3.3e‐05
Intronic	Muscle	301	4544	65	13.2 (7–20)	4.88***	3.3e‐05
Intronic	Gills	301	3821	63	15.1 (9–22)	4.17***	3.3e‐05
ProtChanges	Liver	37	3620	9	2.3 (0–5)	3.99***	8.6e‐05
ProtChanges	Gills	37	3821	12	3.2 (0–7)	3.77***	3.3e‐05
ProtChanges	Muscle	37	4544	8	2.7 (0–6)	2.94**	0.0022
ProtChanges	Brain	37	1428	3	1.1 (0–3)	2.74	0.1024
ProtChanges	Gonads	37	197	0	0.2 (0–1)	0	1.0

*Note:* ‘CI: Low’ and ‘CI:High’ denote the 95% confidence interval bounds for the expected overlap. ‘Fold’ represents the ratio of observed to expected overlap, with asterisks indicating empirical significance levels (*p* values from 50,000 randomizations; ***FDR < 0.001, **FDR < 0.01). Full summary of overlapping genes in Table S5.

Genes harbouring candidate SNPs in intronic regions showed even stronger enrichment, between 4.2 and 5.1‐fold across all five tissues (Table 1, Figure 4). Genes with non‐synonymous candidate SNPs were also significantly enriched for differential expression in liver, gills and muscle (2.9 to 4.0‐fold; Table 1). The large number of differentially expressed genes together with the observed significant enrichment of selection signals in intronic variants suggests an important contribution of regulatory changes driving this rapid adaptive response.

### 3.6 Candidate Genes Are Enriched for Developmental, Metabolic and Cellular Processes

To investigate the biological functions of the genes under selection, we performed a Gene Ontology (GO) enrichment analysis on the 1155 candidate genes located within the genomic window ±25 kb of a SNP surpassing the empirical selection threshold. In parallel we performed GO enrichment on genes both identified as candidate genes and differentially expressed shown to be differentially expressed in Lake Malombe males to characterize the functional landscape of transcriptional response. Full enrichment results are provided in Table S3.

**FIGURE 5:**
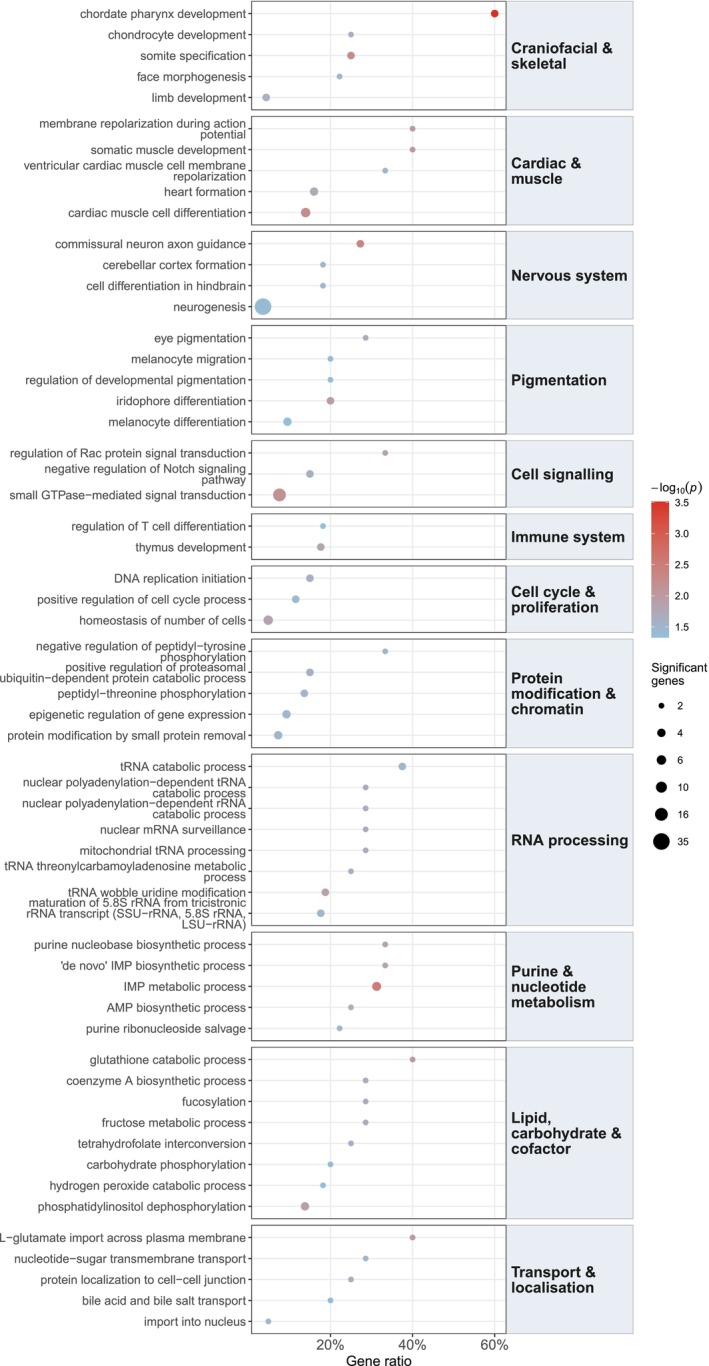
Gene Ontology enrichment of 1155 candidate genes. Dot plot showing significantly enriched Biological Process GO terms among genes ±25 kb of a SNP surpassing the empirical selection threshold. Each dot represents one GO term, grouped into manually curated functional clusters. Dot size reflects the number of significant genes annotated to that term; dot colour indicates statistical significance (−log_10_ Fisher's exact test *p*‐value). The *x*‐axis shows the gene ratio (significant genes annotated to the term/total genes annotated to the term). Only terms with Fisher's exact test *p* < 0.05 are shown.

The analysis of all candidate genes identified 58 significantly enriched GO categories, which were manually grouped into 12 functional clusters based on simplifyEnrichment (Gu and Hübschmann 2023) and GOslim associations (Ashburner et al. 2000; Gene Ontology Consortium 2026). Functional clusters spanned a broad range of processes, from developmental (*nervous system development*, *cardiac & muscle development*, *craniofacial & skeletal patterning*, *pigmentation*) to cellular and metabolic (*cell cycle & proliferation*, *purine & nucleotide metabolism*, *RNA processing*, *lipid, carbohydrate & cofactor metabolism*). A subset of GO terms was significantly enriched in both candidate and DEA gene sets, including *small GTPase‐mediated signal transduction*, *epigenetic regulation of gene expression*, *eye pigmentation*, *chondrocyte development* and *DNA replication initiation*, suggesting that these processes are under both selective pressure and active transcriptional regulation in the Lake Malombe population (Figure 5).

## 4 Discussion

In this study, we investigated the genomic basis of fisheries‐induced evolution in the heavily harvested Lake Malombe population of *Copadichromis mloto* , which has shown a dramatic reduction in size at maturity over the past five decades, consistent with most individuals not surviving to their second breeding season. By leveraging an exceptionally large whole‐genome dataset from almost 200 individuals, we uncovered compelling, widespread evidence for recent positive selection specific to this population. The genomic footprint, characterized by hundreds of outlier loci enriched for functional mutations and genes involved in key developmental pathways strongly suggests a rapid, polygenic adaptive response to the intense harvesting pressure. These findings provide insight into FIE in a tropical freshwater system and stand in contrast to studies in other systems where clear genomic targets of selection have remained elusive (Hutchings and Kuparinen 2021; Pinsky et al. 2021).

A key challenge in studies of wild populations is to unequivocally link genomic signals of selection to a single environmental driver. The genomic divergence of the Lake Malombe population has occurred over its entire history, and we cannot completely disentangle selection exerted by the last 50 years of intense fishing from longer‐term adaptation to the lake's unique environment. Lake Malombe is shallower, more productive and more turbid than Lake Malawi. Furthermore, geological evidence suggests the lake basin may have been completely dry as recently as 200 years ago, consistent with any Malombe‐specific adaptation being a geologically recent phenomenon. These environmental factors have undoubtedly exerted their own selective pressures on traits such as vision, metabolism and immunological pathways. Some of the selection signals we observe may therefore reflect adaptation to these environmental features.

However, several lines of evidence suggest that intense fishing is a primary driver of the specific patterns we observed. First, the most prominent phenotypic shift—a dramatic reduction in size and age at maturity—occurred precisely during the period of fishing intensification. Second, our GO enrichment analysis points specifically at the developmental machinery governing anatomical structure patterning, muscle and heart development, nervous system development as well as cell proliferation and metabolism pathways. While increased water turbidity in Lake Malombe could explain selection on vision‐related genes, and immune system development (Figure 5, Table S3), it is a less parsimonious explanation for a coordinated, genome‐wide signal targeting the core pathways of organismal growth and cellular metabolism. Given that life‐history traits are at least partially heritable (e.g., see Therkildsen et al. (2019) for empirical evidence), an evolutionary response is expected under the extreme mortality imposed by intense fishery. Therefore, we conclude that while the Malombe population has been shaped by multiple factors, the widespread signatures of selection on growth and developmental pathways are compelling candidates for the footprint of recent fisheries‐induced evolution. Future studies could aim to quantify the heritability of these life‐history traits, leveraging the fact that—in contrast to most marine and temperate FIE targets—cichlid fishes are generally easy to breed in captivity and have relatively short generation times.

The widespread distribution of outlier loci across the genome strongly indicates a polygenic architecture of adaptation in the Lake Malombe population. This aligns with findings from experimental FIE systems, where parallel phenotypic responses to size selection were primarily driven by small allele frequency shifts across many genes (Therkildsen et al. 2019). The selective pressure in Lake Malombe, however, is distinct. The indiscriminate ‘Nkatcha’ fishery imposes high mortality without regard to size, a regime predicted by life‐history theory to favour a fundamental shift in energy allocation away from long‐term growth and towards rapid, early‐life reproduction—the classic r/K trade‐off (Heino et al. 2015; Michod 1979).

Given the recent and intense nature of this pressure, the observed polygenic response likely reflects selection on standing genetic variation, resulting in numerous incomplete soft sweeps across the genome. Our methodological approach is particularly well‐suited to detect such signals; by contrasting the *H*‐statistic against the sister population, we specifically targeted signatures of recent, Malombe‐specific sweeps, combining this with population differentiation in our FCS score.

Our results further suggest that this adaptation is achieved through a dual mechanism at the molecular level. The strong enrichment of our outlier set for non‐synonymous mutations indicates that direct protein‐coding changes are a key component of the adaptive response. Simultaneously, the significant enrichment of candidate genes among those that are differentially expressed points to the critical role of regulatory evolution. This regulatory signal was particularly striking in gonad tissue, where candidate genes were almost five‐fold more likely to be differentially expressed than expected by random association. This provides a direct link between the selected loci and the organs central to reproductive timing and reinforces the hypothesis that fishing has selected for an accelerated life history. Exploring expression patterns at different life history stages and in female ovaries and other tissue in future studies may allow for a comprehensive understanding of the regulatory control and biological process involved in early maturation.

A widespread polygenic signal does not preclude the existence of individual loci with larger effects. For complex traits, the underlying genetic architecture is often characterized by a distribution of effect sizes, with many genes of small effect and a tail of a few genes with more substantial impacts. This ‘mixed model’ of adaptation is powerfully illustrated by the above‐mentioned experimental evolution in Atlantic silversides (Therkildsen et al. 2019). While a parallel polygenic response was observed across all replicate lines, some lines also featured strong, idiosyncratic selective sweeps on large chromosomal blocks, demonstrating that both modes of evolution can occur simultaneously in response to the same pressure. Similarly, in wild Eastern Baltic cod, a combined association and selection scan approach successfully identified several key loci under strong directional selection contributing to changes in growth, even against a complex genetic background (Han et al. 2025).

Our findings in *C. mloto* are consistent with this mixed architecture. Against the backdrop of the widespread polygenic signal we detected, our analysis also identified prominent FCS peaks that likely represent the loci with the largest effects on FIE‐related traits in this population. Among the ten most prominent FCS peaks, several genes emerge as compelling candidates with established roles in pathways directly relevant to FIE. In particular, *mpped2a*, *kitlga, kat6a* and *ENSACLG00000001162 (stra6)*, have functions linked to the timing of reproductive development. In humans, the region containing *mpped2a* is associated with puberty timing as the gene is understood to influence the development of gonadotropin‐releasing hormone (GnRH) neurons, which are central to the neuroendocrine control of maturation via the HPG axis (Laisk et al. 2018; Lund et al. 2020). Even more directly, *kitlga* is a well‐characterized regulator of oocyte growth and meiotic arrest in zebrafish (Yao and Ge 2013, 2015). *Kat6a*, a histone acetyltransferase, regulates gonadal development and primordial follicles activation in mammals (Zhang et al. 2025), providing an epigenetic route through which selection could accelerate the onset of maturity. Likewise, *stra6*, which encodes a receptor mediating retinol uptake and retinoic acid signalling, could influence gametogenesis and developmental rate through altered retinoid metabolism, a pathway known to affect both oocyte development and puberty onset in teleosts (Medina et al. 2019; Pradhan and Olsson 2015). Notably, independent support for the importance of neuroendocrine regulation of maturation comes from the set of genes carrying non‐synonymous candidate SNPs. Among these, *npffr1*, encoding the receptor for gonadotropin‐inhibitory hormone (GnIH), harbours a candidate C → T missense variant on chromosome 12 (position chr12:5,310,593). GnIH acts as a conserved inhibitor of gonadotropin release across vertebrates, suppressing the HPG axis by directly targeting GnRH neurons via npffr1 (Dufour et al. 2020; Son et al. 2019). A role that has been demonstrated in cichlid fish specifically (Di Yorio et al. 2016). A functional change in npffr1 could therefore shift the threshold for reproductive activation in the Malombe population. The convergence of protein‐level selection on *npffr1* with the regulatory signal at the *mpped2a* locus, both targeting the same neuroendocrine axis strengthens the hypothesis that accelerated HPG axis activation is a primary molecular target of fisheries‐induced selection in this population. Together, these genes suggest that selection in the Lake Malombe population may have acted not only on specific reproductive regulators but also on higher‐order epigenetic and signalling processes that coordinate the timing of maturation.

Complementing this, other top candidates are directly involved in somatic growth. The gene *zdhhc13*, for example, is a palmitoyltransferase whose disruption in mice leads to severe postnatal growth retardation (Song et al. 2014). Furthermore, ENSACLG00000021917 (*myh7*), a slow‐muscle myosin heavy chain, is essential for muscle development, and its expression level is correlated with growth rate in other teleost fish, including closely related tilapia cichlids (Liu et al. 2020). Additionally, *csrp3* is a key regulator of myogenesis and muscle fibre integrity supporting the link between growth rate and muscle development. Finally, *gnb1l* has been associated with increased body weight and feed efficiency in chickens (Ren et al. 2020), suggesting a conserved role in regulating somatic growth and metabolic performance across vertebrates. Selection on these genes could therefore directly underpin the reduction in adult body size.

Beyond the classic life‐history shifts in growth and maturation, intense harvesting is expected to select for other traits that reduce capture probability, such as changes in fecundity or the evolution of avoidance behaviours (Heino et al. 2015). While we did not measure these traits directly, our genomic results provide intriguing clues that such selection may be occurring. The strong enrichment of candidate genes involved in high‐level nervous system development—including terms such as neurogenesis and commissural neuron axon guidance—suggests that selection is actively shaping the neural architecture of the Lake Malombe population. Such changes could plausibly underpin the evolution of adaptive behaviours like increased wariness or altered shoaling. This behavioural component may also be linked to our top candidate genes; for instance, selection on the slow‐muscle myosin gene ENSACLG00000021917 (*myh7*) could not only influence growth but also modulate the power and endurance of the flight response, a critical component of net avoidance. Together, this suite of candidate genes connects the genomic signal to a plausible biological response and provides promising targets for functional follow ups to establish the mechanistic links between the intense fishing pressure in Lake Malombe and the observed evolution of these key physiological traits.

The potential for such traits to evolve in this system is high. For example, recent work has documented significant variation in physiological and behavioural traits, including swimming performance and exploration, both within Lake Malombe *C. mloto* and compared to closely related species (Sawasawa et al. 2025). This suggests that heritable variation is present in these traits, making an evolutionary response to selection within *C. mloto* highly plausible. Future studies, leveraging the experimental tractability of the system, could directly test for divergent behavioural syndromes and other traits like fecundity between the heavily‐ and weakly‐fished populations and experimentally map their genetic basis.

In conclusion, our study revealed a widespread, polygenic signal of recent selection in an exceptionally heavily fished population of *C. mloto* . The concentration of this genomic footprint on the developmental pathways that govern growth and maturation makes a strong argument that this represents an adaptive response to the intense harvesting pressure. The suite of candidate genes we identified provides promising targets for functional studies, while the biology of this system presents a unique opportunity to experimentally dissect the genomic basis of affected traits and their evolutionary potential, a task intractable for most marine fisheries targets.

From a management perspective, our findings present a complex picture. The predominance of soft, incomplete sweeps on standing variation suggests that much of the original genetic diversity has been maintained. However, the highly polygenic nature of the response means that reversing these widespread genetic shifts may be slow and difficult, potentially creating a ‘Darwinian debt’ that could compromise the stock's long‐term resilience (Pandolfi 2009). Therefore, monitoring the evolutionary trajectory of the population under future management interventions will be crucial for understanding and mitigating the long‐term consequences of human‐induced evolution.

## Author Contributions

A.H.H. and H.S. conceived the study. H.S. and B.R. collected samples. A.H.H. performed all analyses with contributions from F.C.J., J.C.G., and H.S. G.B. and H.S. provided supervision. A.H.H. and H.S. wrote the manuscript. All Authors approved the final manuscript.

## Funding

This work was supported by Fonds Wetenschappelijk Onderzoek, 11E0623N, G047521N. Cambridge‐Africa ALBORADA Research Fund.

## Conflicts of Interest

The authors declare no conflicts of interest.

## Supporting information


**Figure S1:** Quantile–quantile plot of empirical Fisher's Combined Score (FCS) values against FCS values from neutral demographic simulations. Deviation above the 1:1 line (solid red) indicates an excess of high‐scoring loci relative to neutral expectation. The dashed red line marks the empirically derived selection threshold (FCS = 5.51), above which SNPs are classified as selection outliers.
**Figure S2:** Detailed plot of Fisher's combined score (yellow), Population Branch statistic (blue) and *H*‐score (green) and gene positions of a major FCS peak on chr2: 11606365–11,656,365.
**Figure S3:** Detailed plot of Fisher's combined score (yellow), Population Branch statistic (blue) and *H*‐score (green) and gene positions of a major FCS peak on chr3: 3326518–3,376,518.
**Figure S4:** Detailed plot of Fisher's combined score (yellow), Population Branch statistic (blue) and *H*‐score (green) and gene positions of a major FCS peak on chr6: 6472995–6,582,963.
**Figure S5:** Detailed plot of Fisher's combined score (yellow), Population Branch statistic (blue) and *H*‐score (green) and gene positions of a major FCS peak on chr7:10285777–10,338,291.
**Figure S6:** Detailed plot of Fisher's combined score (yellow), Population Branch statistic (blue) and *H*‐score (green) and gene positions of a major FCS peak on chr7:44175985–44,244,688.
**Figure S7:** Detailed plot of Fisher's combined score (yellow), Population Branch statistic (blue) and *H*‐score (green) and gene positions of a major FCS peak on chr12:5505879–5,557,210.
**Figure S8:** Detailed plot of Fisher's combined score (yellow), Population Branch statistic (blue) and *H*‐score (green) and gene positions of a major FCS peak on chr12:6036511–6,086,511.
**Figure S9:** Detailed plot of Fisher's combined score (yellow), Population Branch statistic (blue) and *H*‐score (green) and gene positions of a major FCS peak on chr20:8335345–8,385,356.
**Figure S10:** Detailed plot of Fisher's combined score (yellow), Population Branch statistic (blue) and *H*‐score (green) and gene positions of a major FCS peak on chr23:20791821–20843857.

**Table S1:** Metadata of sequenced specimens of *Copadichromis mloto* , used in this study. Including sampling location, sequencing depth, assigned population, *C. mloto* clade & NCBI SRA accession numbers. Individuals also sampled for differential gene expression analysis (RNA‐seq) are indicated.

**Table S2:** Metadata of sequences from Blumer et al. 2025 used in this study. Including Population assignment, Malawi cichlid clade & NCBI SRA accession numbers.

**Table S3:** Output of Gene Ontology test (topGO) for all candidate genes within ±25 kb of outlier SNPs and (ii) the subset of those candidate genes that are differentially expressed between Lake Malombe and Lake Malawi.

**Table S4:** Differential expression results log2 fold change (log2FC) and –log10 (adjusted *p*‐value) in five tissues (Liver, Brain, Muscle, Gonads, Gills).

**Table S5:** Summary table of candidate genes, genes with non‐synonymous and/or intronic variants above defined empirical selection threshold, and differentially expressed genes in five tissues (Liver, Brain, Muscle, Gonads, Gills).

## Data Availability

Raw sequencing data are publicly available in the NCBI Sequence Read Archive (SRA); accession numbers are listed in Table S1. All scripts and analysis pipelines used in this study are archived on Zenodo (https://doi.org/10.5281/zenodo.17649119) to ensure reproducibility. Variant call format (VCF) files are available on Zenodo: (https://doi.org/10.5281/zenodo.17657791). Benefit Sharing: A research collaboration was developed with scientists from Malawi through collaboration with the University of Malawi, and the Department of Fisheries. This research was conducted in full compliance with the Nagoya Protocol on Access and Benefit Sharing. Research permits and material transfer agreements were obtained from the Government of Malawi. The results of research have been shared with the local communities and the broader scientific community and the research addresses a priority concern, in this case the conservation of studied organisms. Benefits arising from this research have been shared through collaboration with Malawian research institutions, training and capacity building and co‐authorship with in‐country scientists. More broadly, our group is committed to international scientific partnerships, as well as institutional capacity building.
