# Supplementary material for: Recent Polygenic Adaptation in Heavily Fished Malawi Cichlids
Source: Mol Ecol. 2026 Jun 22;35(12):e70443. doi: 10.1111/mec.70443 (PMC13284802; doi:10.1111/mec.70443)
Supplement: Supplementary file 1 — Figure S1: Quantile–quantile plot of empirical Fisher's Combined Score (FCS) values against FCS values from neutral demographic simulations. Deviation above the 1:1 line (solid red) indicates an excess of high‐scoring loci relative to neutral expectation. The dashed red line marks the empirically derived selection threshold (FCS = 5.51), above which SNPs are classified as selection outliers. Figure S2: Detailed plot of Fisher's combined score (yellow), Population Branch statistic (blue) and H‐score (green) and gene positions of a major FCS peak on chr2: 11606365–11,656,365. Figure S3: Detailed plot of Fisher's combined score (yellow), Population Branch statistic (blue) and H‐score (green) and gene positions of a major FCS peak on chr3: 3326518–3,376,518. Figure S4: Detailed plot of Fisher's combined score (yellow), Population Branch statistic (blue) and H‐score (green) and gene positions of a major FCS peak on chr6: 6472995–6,582,963. Figure S5: Detailed plot of Fisher's combined score (yellow), Population Branch statistic (blue) and H‐score (green) and gene positions of a major FCS peak on chr7:10285777–10,338,291. Figure S6: Detailed plot of Fisher's combined score (yellow), Population Branch statistic (blue) and H‐score (green) and gene positions of a major FCS peak on chr7:44175985–44,244,688. Figure S7: Detailed plot of Fisher's combined score (yellow), Population Branch statistic (blue) and H‐score (green) and gene positions of a major FCS peak on chr12:5505879–5,557,210. Figure S8: Detailed plot of Fisher's combined score (yellow), Population Branch statistic (blue) and H‐score (green) and gene positions of a major FCS peak on chr12:6036511–6,086,511. Figure S9: Detailed plot of Fisher's combined score (yellow), Population Branch statistic (blue) and H‐score (green) and gene positions of a major FCS peak on chr20:8335345–8,385,356. Figure S10: Detailed plot of Fisher's combined score (yellow), Population Branch statistic (blue) and H‐score (green) and gene po [file MEC-35-e70443-s006.docx]

**Supplemental Information for:**

**Recent polygenic adaptation in heavily fished Malawi cichlids**

Alexander Hooft van Huysduynen^1,2^, Francisco Campuzano Jiménez^1^, Julia Camacho Garcia^1^, Gudrun De Boeck^2^, Bosco Rusuwa^3^, Hannes Svardal^1,4^

1) Evolutionary Ecology Group, Department of Biology, University of Antwerp, 2020 Antwerp, Belgium,

2) ECOSPHERE, Department of Biology, University of Antwerp, 2020 Antwerp, Belgium,

3) School of Natural and Applied Sciences, University of Malawi, Zomba, Malawi.

4) Naturalis Biodiversity Center, 2333 Leiden, The Netherlands,

**Supplementary Figure S1**

*Supplementary Figure S1: Quantile–quantile plot of empirical Fisher's Combined Score (FCS) values against FCS values from neutral demographic simulations. Deviation above the 1:1 line (solid red) indicates an excess of high-scoring loci relative to neutral expectation. The dashed red line marks the empirically derived selection threshold (FCS = 5.51), above which SNPs are classified as selection outliers.*

**Supplementary Figure S2**

*Supplementary Figure S2: Detailed plot of Fishers combined score (yellow), Population Branch statistic (blue) and H-score (green) and gene positions of a major FCS peak on chr2: 11606365 – 11656365.*

**Supplementary Figure S3**

*Supplementary Figure S3: Detailed plot of Fishers combined score (yellow), Population Branch statistic (blue) and H-score (green) and gene positions of a major FCS peak on chr3: 3326518 – 3376518.*

**Supplementary Figure S4**

*Supplementary Figure S4: Detailed plot of Fishers combined score (yellow), Population Branch statistic (blue) and H-score (green) and gene positions of a major FCS peak on chr6: 6472995 – 6582963.*

**Supplementary Figure S5**

*Supplementary Figure S5: Detailed plot of Fishers combined score (yellow), Population Branch statistic (blue) and H-score (green) and gene positions of a major FCS peak on chr7:10285777 – 10338291.*

**Supplementary Figure S6**

*Supplementary Figure S6: Detailed plot of Fishers combined score (yellow), Population Branch statistic (blue) and H-score (green) and gene positions of a major FCS peak on chr7:44175985 – 44244688.*

**Supplementary Figure S7**

*Supplementary Figure S7: Detailed plot of Fishers combined score (yellow), Population Branch statistic (blue) and H-score (green) and gene positions of a major FCS peak on chr12:5505879 – 5557210.*

**Supplementary Figure S8**

*Supplementary Figure S8: Detailed plot of Fishers combined score (yellow), Population Branch statistic (blue) and H-score (green) and gene positions of a major FCS peak on chr12:6036511-6086511.*

**Supplementary Figure S9**

*Supplementary Figure S9: Detailed plot of Fishers combined score (yellow), Population Branch statistic (blue) and H-score (green) and gene positions of a major FCS peak on chr20:8335345 – 8385356.*

**Supplementary Figure S10**

*Supplementary Figure S10: Detailed plot of Fishers combined score (yellow), Population Branch statistic (blue) and H-score (green) and gene positions of a major FCS peak on chr23:20791821 – 20843857.*
